# INVA8001, a novel and highly selective chymase inhibitor, ameliorates liver inflammation, fibrosis, and hyperplasia in Mdr2 knockout mice

**DOI:** 10.3389/fmed.2026.1840071

**Published:** 2026-06-09

**Authors:** Sameer Sharma, Lixian Chen, Tianhao Zhou, Meenakshi Chawla, Anita Ganjoo, Shunichiro Okada, Salvatore Alesci, Krishnan Nandabalan, Heather Francis

**Affiliations:** 1 AlphaMeld Corporation, Guilford, CT, United States; 2 Invea Therapeutics, Inc., Guilford, CT, United States; 3 Department of Structural and Cellular Biology, Tulane University School of Medicine, New Orleans, LA, United States; 4 Indiana University School of Medicine, Indianapolis, IN, United States; 5 Scientific Consultant, Indianapolis, IN, United States

**Keywords:** biliary senescence, chymase inhibitor, ductular reaction, fibrosis, liver inflammation, mast cell chymase, mast cell inhibitor, primary sclerosing cholangitis

## Abstract

**Aims:**

Mast cells (MCs) play a significant role in autoimmune diseases by mediating inflammatory responses, innate and adaptive responses, angiogenesis, and various pathological processes. In chronic liver diseases, MC numbers are significantly increased, and their degranulation leads to release of mediators, including chymase, which increases inflammation, activates hepatic stellate cells (HSCs), leading to fibrosis. Primary sclerosing cholangitis (PSC) is a chronic, progressive cholestatic liver disease characterized by inflammation, fibrosis, ductular reaction, and eventual liver failure. Although MCs and chymase have been implicated in PSC pathogenesis, therapeutic targeting of chymase remains largely unexplored. In this study, we examined the efficacy of INVA8001, a highly selective and potent small-molecule chymase inhibitor in the Mdr2 knockout mouse (*Mdr2^−/−^*) model of PSC.

**Methods:**

We evaluated chymase and other MC markers in liver biopsy samples from late-stage PSC patients. The effect of chymase inhibition was evaluated using INVA8001 in *Mdr2^−/−^* mice. Ten-week-old male *Mdr2^−/−^* mice were injected intraperitoneally (IP) with 20 mg/kg of INVA8001 daily for 2 weeks and parameters of disease pathogenesis, including MC activation, inflammation, fibrosis, biliary pathology, and cholestasis were evaluated. Histological, immunohistochemical, biochemical, and molecular analyses were conducted to evaluate the effects of INVA8001 treatment in *Mdr2^−/−^* mice.

**Results:**

Liver biopsies from explanted PSC patients showed increased numbers of chymase-positive cells compared with control samples (collected from non-diseased patients). INVA8001 treatment reduced chymase activity and was associated with decreased MC accumulation, inflammation, histological damage, fibrosis, ductular reaction, and biliary senescence in *Mdr2^−/−^* mice.

**Discussion:**

The current data show the pathophysiological role of chymase in PSC and the impact of a selective chymase inhibitor on PSC disease pathogenesis. These findings may have broader implications for MC-driven diseases such as chronic urticaria, atopic dermatitis, asthma, metabolic dysfunction-associated steatohepatitis (MASH), and eosinophilic gastrointestinal diseases, among others, where chymase is central to the disease pathogenesis.

**Conclusion:**

Our findings suggest that chymase is strongly associated with PSC pathogenesis and that INVA8001 may represent a promising new therapeutic candidate for hepatobiliary disorders, including PSC. Chymase inhibition simultaneously targets MC activation, inflammation, fibrosis, and biliary senescence, and offers a multifaceted approach to treating PSC and other MC-related disorders.

## 1 Introduction

Primary sclerosing cholangitis (PSC) is a rare, chronic cholestatic disease that gradually progresses from early to end-stage liver disease, and detection often occurs at late-stage, thus necessitating liver transplantation for patient survival. In the United States, there are currently no Food and Drug Administration (FDA)-approved treatments for PSC management. PSC is primarily characterized by liver inflammation and fibrosis, along with the destruction of the intra- and extrahepatic bile ducts leading to multiple strictures in the biliary tree and eventually cirrhosis. According to 2021 data, the incidence and prevalence of PSC in the general population was 0.75 (95% CI: 0.542–1.05) and 11.16 (95% CI: 7.78–16.02) per 100,000 people, respectively (1). The incidence rate of PSC has significantly increased over time and approximately 40% of patients with this disease ultimately require liver transplants. While PSC is considered a rare disorder, it is associated with high morbidity and mortality, as prognosis is often poor and the condition markedly increases the risk of developing cholangiocarcinoma (2).

Mast cells (MCs) are critical cellular components of the immune system, playing a key role in maintaining tissue homeostasis, by regulating epithelial function and integrity, modulating both innate and adaptive mucosal immunity, and mediating local neuro-immune interactions. Substantial evidence from both human and animal models has validated the role of MCs and their mediators in immune-mediated cholestatic disorders, such as PSC. MCs positive with both tryptase, a MC–specific serine protease and canonical marker of MC activation, and c-KIT, the receptor for stem cell factor (SCF) ligand (bioactive, soluble SCF), required for MC development and survival, infiltrate the fibrotic portal tracts in the livers of PSC patients and their numbers have been positively correlated with disease severity (3). It has been shown that, in the early stages of PSC, MCs predominantly localize around the bile ducts, whereas in the later stages of the disease, MCs are primarily found in the fibrous septa. An increase in MC markers such as c-KIT and the high-affinity IgE receptor, FceR1, and MC mediators such as chymase, tryptase and histamine have been observed in livers from PSC patients, as well as in the genetic murine model of PSC (*Mdr2^−/−^* mice). Furthermore, treatment with cromolyn sodium, a MC stabilizer, in *Mdr2^−/−^* mice attenuates the levels of MC mediators, inflammation, tissue damage, and fibrosis (4).

Upon activation by pathogen-associated molecular patterns (PAMPs) and danger-associated molecular patterns (DAMPs), MCs undergo degranulation and release a variety of bioactive mediators including chymase (5). Chymase, a serine protease, plays a pivotal role in the recruitment of various immune cells, such as eosinophils, neutrophils, macrophages, and lymphocytes initiating a cascade of events that promotes inflammation, epithelial barrier dysfunction and fibrosis (6). Once chymase is released, it triggers the activation of several key substrates including angiotensin II (Ang-II), matrix metalloproteinases (MMP-1, MMP-9), transforming growth factor beta (TGF-*β*), collagen I, and chemokines such as interleukin-33 (IL-33) (6–11). In addition, chymase leads to the further proliferation of MCs by enzymatically converting SCF to its bioactive soluble form, which in turn binds to c-KIT receptors on MCs and supports proliferation, migration, survival, and differentiation of MCs (12, 13). In chronic liver injury, the expression of SCF is induced in biliary epithelial cells that line bile ducts showing periductal fibrosis and inflammation as compared to non-affected bile ducts, suggesting that reactive cholangiocytes may play a role in MC accumulation and proliferation (14).

Chymase activity is induced in inflamed livers of hamsters fed with a methionine- and choline-deficient (MCD) diet and blocking chymase function inhibits Ang-II and MMP-9 activity, as shown in liver biopsies from hamsters fed with MCD diet (15). Additionally, chymase inhibition alleviates steatohepatitis, limits infiltration of MCs, decreases expression of pro-inflammatory and pro-fibrotic mediators, and reduces fibrosis in the liver in various steatohepatitis animal models (16). Although elevated levels of chymase have been observed in liver samples from PSC patients, there is no published literature to date examining the effect of chymase inhibition on PSC pathogenesis.

**Figure 1:**
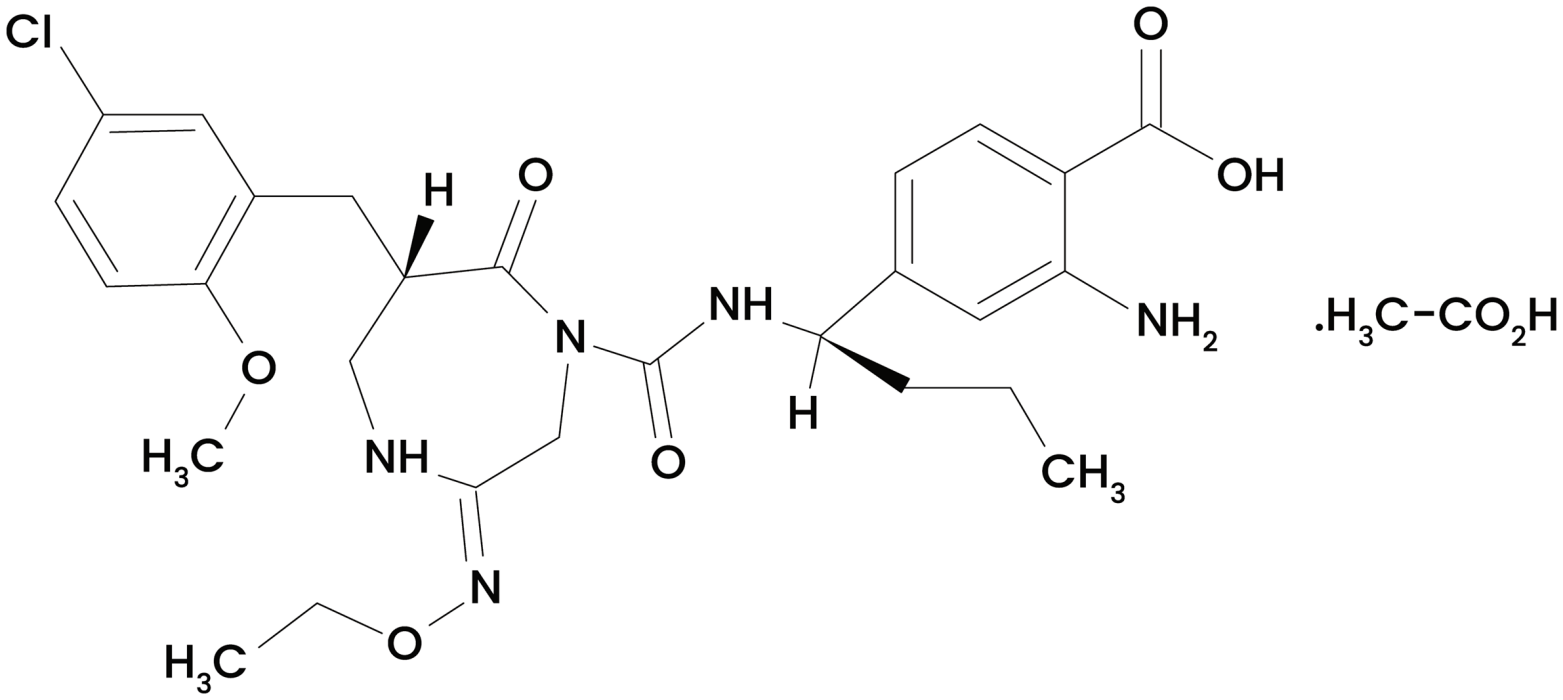
Structure of INVA8001, a novel chymase inhibitor. Molecular weight: 620 g/mol. Refer to Table 1 for selectivity data.

INVA8001 (formerly ASB17061; in-licensed by Invea Therapeutics, Inc. from Daiichi Sankyo Company, Ltd.) is a complex organic molecule with a benzene ring substituted with a side chain containing a butyl group and a 4-diazepan-1-yl ring (a 7-membered ring containing nitrogen) with an attached chloro-methoxybenzyl group (Figure 1). The compound is crystallized with acetic acid molecules which are incorporated into its structure as solvent molecules. INVA8001 is an orally administered, highly potent and selective chymase inhibitor with a high safety index. *In vitro* primary pharmacology studies demonstrate that INVA8001 is a potent and highly selective inhibitor of MC-chymase and shows remarkable selectivity over related enzymes (Table 1) (17).

**Table 1:** Selectivity of INVA8001.

Read out(s)	Results (IC50)
Mouse mast cell protease 4 (mMCP-4)	0.03 μM
Human chymase	0.02 μM
Bovine α-chymotrypsin	3.4 μM
Human cathepsin G	32.1 μM
Bovine trypsin	>100 μM
Human elastase	>100 μM

The present study was designed to investigate the effect of a highly selective chymase inhibitor, INVA8001 on the progression of PSC.

## 2 Materials and methods

### 2.1 Materials

Study drug, INVA8001 (ASB17061), was supplied as a gift sample by Daiichi Sankyo Company, Ltd. with >95% purity. Reagents were purchased from Sigma-Aldrich (St. Louis, MO) unless otherwise indicated. Information for all antibodies used is described in Supplementary Table 1. The Quick-RNA Miniprep Plus Kit for RNA isolation was purchased from Zymo Research (Irvine, CA). Selected mouse primers were purchased from Qiagen (Valencia, CA). The iScript cDNA Synthesis Kit (170-8891) and iTaq Universal SYBR Green Supermix were purchased from Bio-Rad (Hercules, CA). The information on real-time PCR (qPCR) primers used is listed in Supplementary Table 2.

### 2.2 Animal models

Animal procedures were performed according to protocols approved by Indiana University School of Medicine Institutional Animal Care and Use Committee. Ten-week-old male *Mdr2^−/−^* mice (*n* = 8) were treated with daily IP injections of INVA8001 (formulated in 0.5% methyl cellulose and dosed at 20 mg/kg) for 2 weeks. Male *Mdr2^−/−^* mice (*n* = 8) were collected at twelve-weeks for PSC model. Twelve-week-old male Friend Virus B NIH Jackson (FVB/NJ [FVB/wild type (WT) control], *n* = 8) mice were used as control WT for *Mdr2^−/−^*.

In all groups, serum, liver, cholangiocytes, and cholangiocyte supernatants were collected as described (18, 19). The collected liver was divided into three portions: (i) snap-frozen tissue; (ii) OCT-embedded blocks (5 μm cryosections) for immunofluorescence (IF); and (iii) formalin-fixed, paraffin-embedded (FFPE) blocks (4 μm sections) for immunohistochemistry (IHC).

### 2.3 Human samples

Human explant liver tissues from both male and female patients were collected from either normal controls (liver *n* = 5) or late-stage PSC patients (liver *n* = 5) under the Institutional Review Board (IRB) approved protocol at Indiana University School of Medicine (IUSM). PSC liver tissues were collected from explanted livers at the time of liver transplantation and were therefore classified as transplant-stage/end-stage PSC. The PSC patients included in this analysis had no documented inflammatory bowel disease and were not receiving PSC-specific medication at the time of tissue collection. Informed consent was collected from patients for the human specimens that were utilized in this study. An additional three healthy control liver samples were purchased from Sekisui Xeno Tech (Kansas City, KS). The samples were stained by IHC for chymase and tryptase in FFPE liver sections.

### 2.4 Evaluation of liver damage, ductular reaction and biliary senescence

Liver damage was assessed by hematoxylin and eosin (H&E) staining in FFPE mouse liver sections and scored in a coded fashion to determine portal inflammation. Serum and liver total bile acids (TBAs) were determined using a TBA Assay Kit (Colorimetric, Sigma-Aldrich).

Ductular reaction was evaluated in FFPE mouse liver sections by IHC for CK-19 to specifically mark bile ducts. Stained slides were scanned by a digital scanner (Aperio AT2 scanner; Leica Biosystems). CK-19-positive area was quantified using the Image-Pro Premier software (Rockville, MD). Biliary senescence was evaluated by: (i) IF for p16 expression in frozen liver sections co-stained with CK-19 (to mark bile ducts) and (ii) mRNA expression of cellular senescence markers, cyclin dependent kinase inhibitor 2a (*Cdkn2a*; gene for p16), cyclin dependent kinase inhibitor 2c (*Cdkn2c*, gene for p18) and galactosidase, beta 1-like (*Glb1l*) in isolated mouse cholangiocytes (3, 19).

### 2.5 Assessment of liver fibrosis

Collagen deposition was determined by Fast Green/Sirius Red staining in FFPE mouse liver sections and quantified by the Image-Pro Premier software (Rockville, MD). Liver fibrosis was also determined by measuring hydroxyproline levels in total liver samples using the hydroxyproline assay kit (MAK008-1KT; Millipore Sigma, Billerica, MA). To detect activated HSCs, IF for desmin (co-stained with CK-19) was performed in frozen liver sections with semi-quantification (20, 21). The mRNA expression of the fibrosis markers, collagen type I alpha 1 (*Col1a1*) and tissue inhibitor of metalloproteinases 2 (*Timp2*) and transforming growth factor beta 1 (*Tgfb1*) was measured in total mouse livers by qPCR.

### 2.6 Evaluation of liver inflammation and MC infiltration

Liver inflammation was evaluated by IHC for F4/80 in FFPE mouse liver sections, and positive cells were quantified by Image-Pro Premier software (Rockville, MD). Liver MC infiltration was detected with IHC for tryptase *β*-2 (*Tpsb2*) and mucosal MC chymase-1 (*mMCP1*) in liver sections. MCs were semi-quantified as *Tpsb2* or *mMCP1* positivite cell number/total area with Image-Pro Premier software (Rockville, MD). MC activation was determined by qPCR for Fc fragment of IgE, high affinity I, receptor for alpha polypeptide (*Fcer1a*), *Mcpt1* and *Tpsb2* in total liver samples.

### 2.7 Statistical analysis

All data are expressed as the mean ± SD. Differences between groups were analyzed by unpaired Student’s *t* test when two groups were analyzed and one-way ANOVA when more than two groups were analyzed, followed by an appropriate *post hoc* test. Statistical analyses and generation of graphs were performed with GraphPad Prism (version 9.2.0; GraphPad Software, LLC; San Diego, CA). The level of significance was set at *p* < 0.05.

## 3 Results

### 3.1 Chymase-positive MCs are elevated in human PSC liver explants

**Figure 2:**
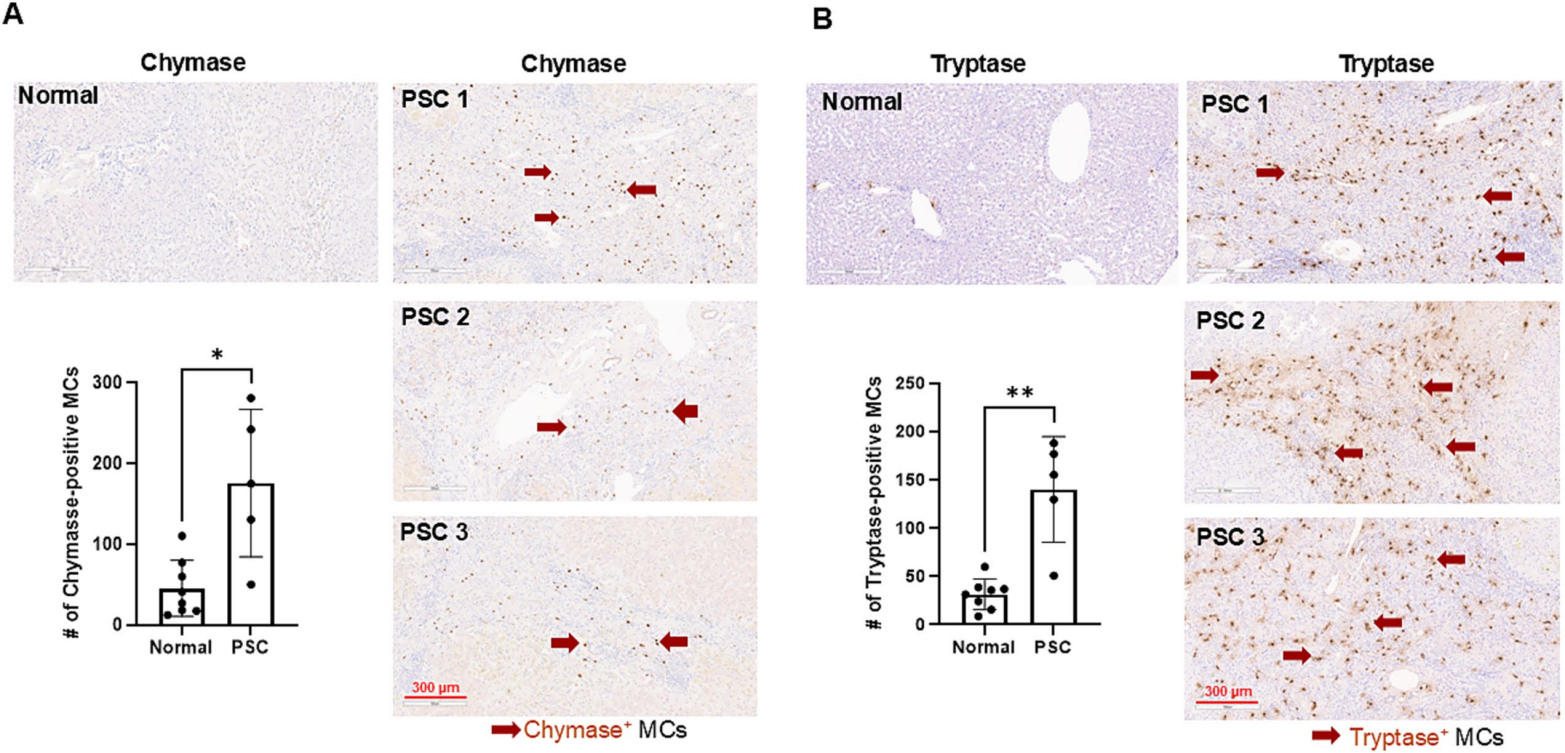
Chymase and tryptase expression in human PSC. **(A)** Representative images for chymase and **(B)** tryptase IHC staining (×10; scale bar, 300 μm) and semi-quantification (average positive number count for three portal images per sample) in human samples. Data are mean ± standard deviation. *n* = 8 human normal control, and *n* = 5 human PSC. * *p* < 0.05; ** *p* < 0.01.

By IHC, we observed a significantly higher number of chymase-positive MCs in PSC patients compared to the controls (Figure 2A). Similarly, a marked increase in the number of tryptase-positive MCs was observed in PSC liver samples compared to healthy controls (Figure 2B).

### 3.2 INVA8001 reduces MC accumulation in liver samples from *Mdr2^−/−^* mice

**Figure 3:**
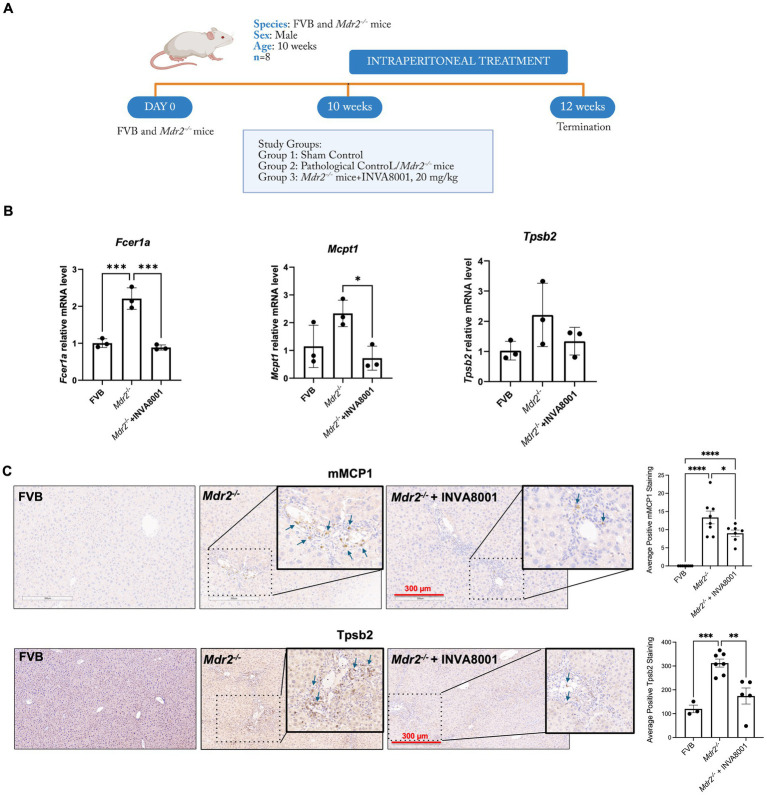
INVA8001 reduces MC accumulation in *Mdr2*^−/−^mice. **(A)** Schematic outline of *in vivo* treatment in FVB and *Mdr2*^−/−^ mice; **(B)** *Fcer1a*, *Mcpt1*, and *Tpsb2* mRNA expression in livers from FVB, *Mdr2*^−/−^, and *Mdr2*^−/−^ mice treated with INVA8001; **(C)** Representative images for *mMCP-1* and *Tpsb2* IHC staining (×10; scale bar, 300 μm) and semi-quantification in FVB, *Mdr2*^−/−^, and *Mdr2*^−/−^ mice treated with INVA8001. Data are mean ± standard deviation. *n* = 5–8 portal images per sample imaged from *n* = 3–8 mice per group for IHC; *n* = 3 for qPCR * *p* < 0.05, ** *p* < 0.01, *** *p* < 0.001, **** *p* < 0.0001.

Since we found increased chymase levels in human PSC livers, we evaluated the pharmacological inhibition of chymase in modulating PSC in *Mdr2^−/−^* mice. INVA8001 was administered at a dose of 20 mg/kg (formulated in 0.5% methyl cellulose) for 2 weeks and injected daily via an intraperitoneal (IP) route of administration (Figure 3A). All treatment groups were compared to *Mdr2^−/−^* mice.

INVA8001 attenuated the expression of *Fcer1a* and *Mcpt1* in the liver samples from *Mdr2^−/−^* mice; however, the expression of *Tpsb2* showed a decreasing trend (not statistically significant) (Figure 3B).

This was further correlated with the histological analysis, which revealed a substantial accumulation of chymase (mMCP1)-positive and tryptase (*Tpsb2*)-positive cells in *Mdr2^−/−^* mice. INVA8001 dosed at 20 mg/kg, IP led to a significant reduction in the numbers of both chymase and tryptase-positive cells (Figure 3C). These results suggest that chymase inhibition effectively reduces MC accumulation in inflamed livers of *Mdr2^−/−^* mice and may mitigate MC-driven effects in PSC.

### 3.3 INVA8001 ameliorates liver damage and inflammation in *Mdr2^−/−^* mice

**Figure 4:**
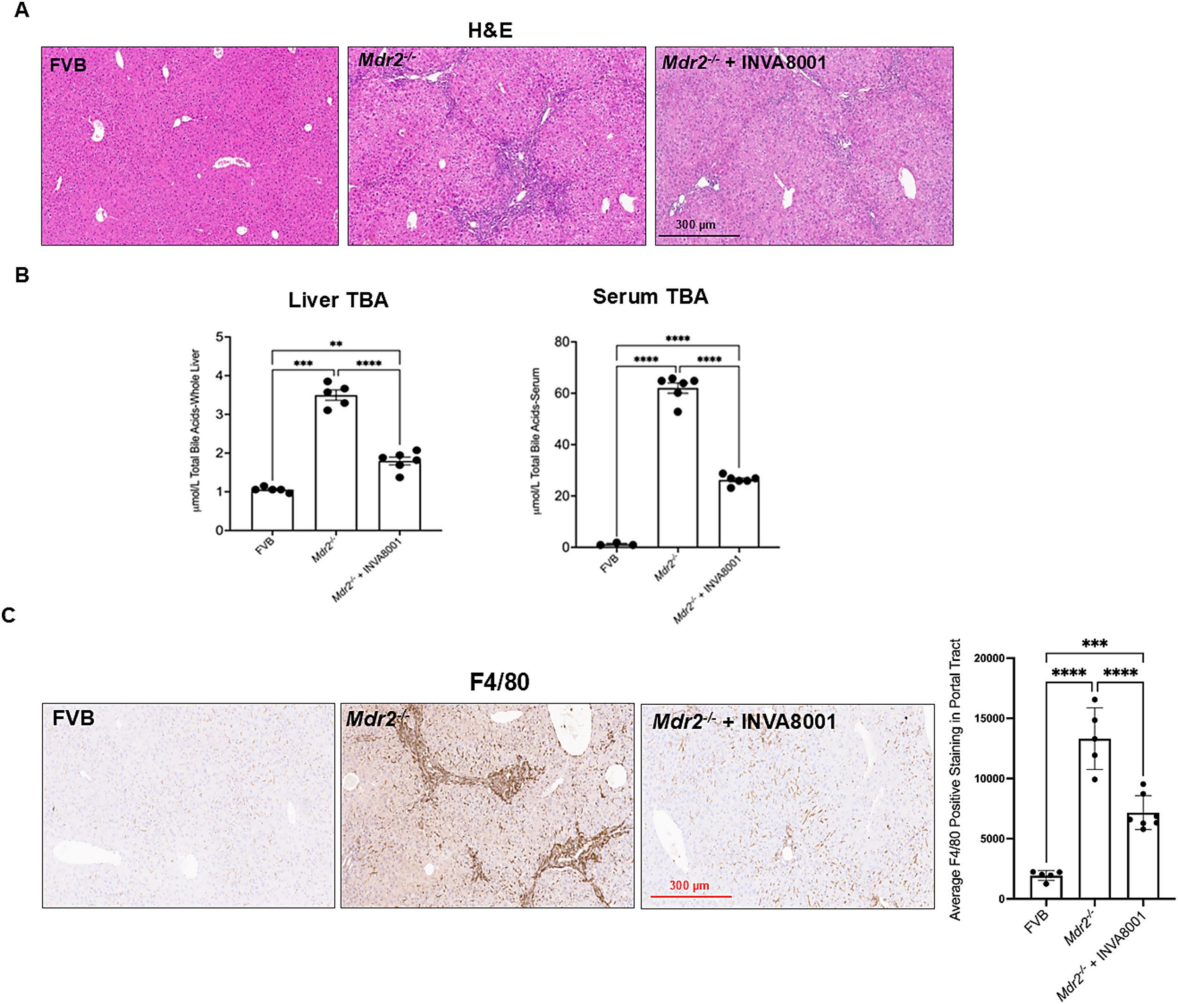
INVA8001 ameliorates liver damage and inflammation in *Mdr2*^−/−^ mice. **(A)** Representative H&E staining (×10; scale bar, 300 μm) in mouse livers; **(B)** Liver (left) and serum (right) TBA levels in FVB, *Mdr2*^−/−^, and *Mdr2*^−/−^ mice treated with INVA8001; **(C)** F4/80 staining (×10; scale bar, 300 μm) and semi-quantification in mouse samples. Data are mean ± standard deviation. *n* = 5–7 portal areas per mouse imaged from *n* = 5–7 mice per group for F4/80. * *p* < 0.05, ** *p* < 0.01, *** *p* < 0.001, **** *p* < 0.0001.

*Mdr2^−/−^* mice exhibit hallmark histological features of PSC such as lobular damage, necrosis, and inflammation, as revealed by H&E staining. INVA8001 at the tested dose showed an amelioration of histological damage and portal inflammation in *Mdr2^−/−^* mice (Figure 4A). Furthermore, TBA levels were shown to be elevated in *Mdr2^−/−^* mice as compared to FVB controls, both in serum and hepatic tissue samples. INVA8001 treatment in *Mdr2^−/−^* mice led to a significant reduction in TBA accumulation at a dose of 20 mg/kg, IP (Figure 4B). Although INVA8001 mitigated bile acid accumulation and hepatic damage, no significant changes in liver enzymes were observed (data not shown). An increased number of macrophages were also evaluated by F4/80 staining in the portal tracts of *Mdr2^−/−^* mice and treatment with INVA8001 significantly reduced the number of infiltrating macrophages (Figure 4C).

### 3.4 INVA8001 attenuates fibrosis in livers from *Mdr2^−/−^* mice

**Figure 5:**
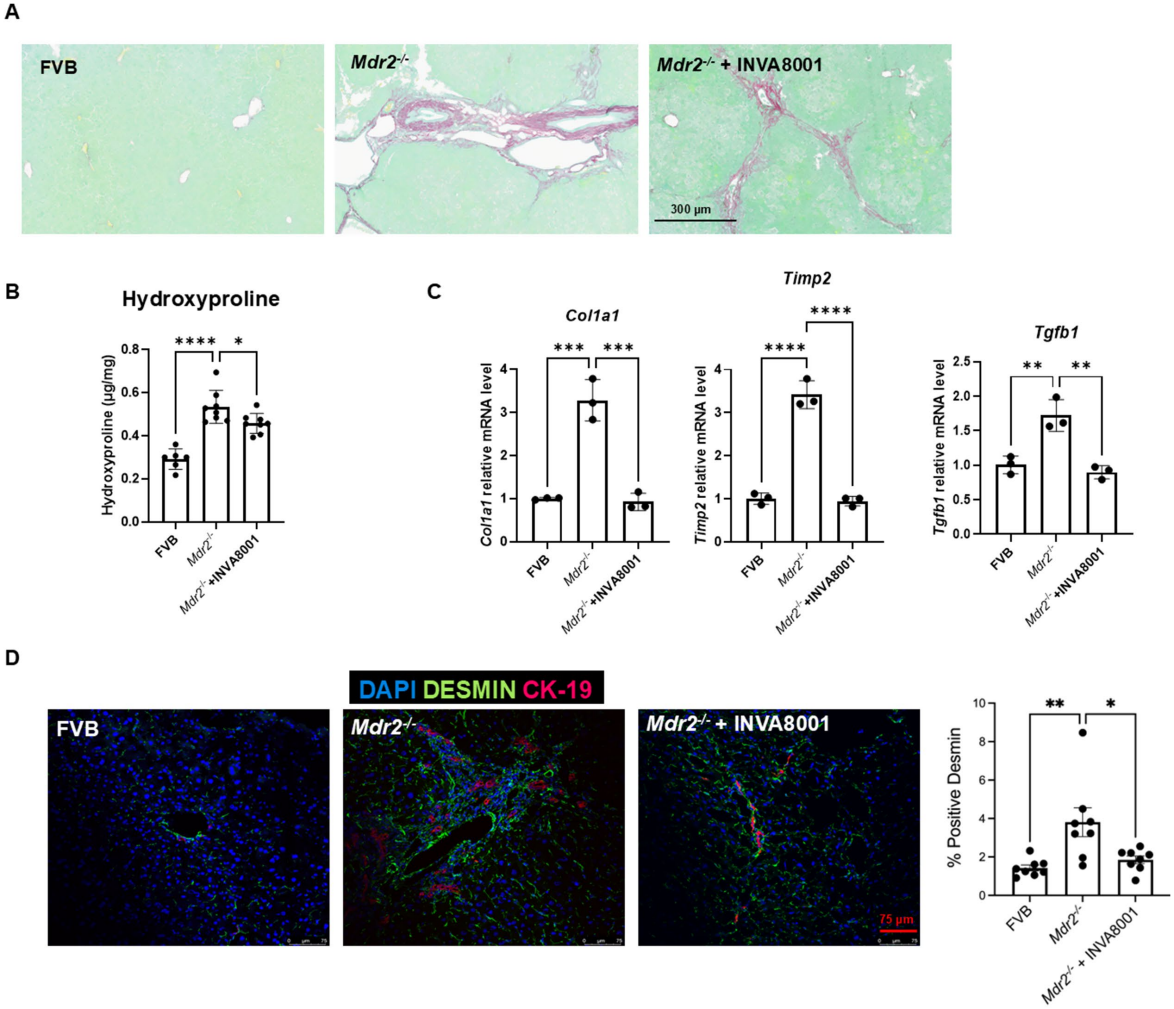
INVA8001 attenuates fibrosis in *Mdr2*^−/−^ mice. **(A)** Representative FG/SR staining (×10; scale bar, 300 μm) in mouse samples; **(B)** Hydroxyproline content in mouse samples (g/mg); **(C)** *Col1a1*, *Timp2*, and *Tgfb1* mRNA expression in total liver from mouse samples; and **(D)** Representative IF images (×20; scale bar, 75 μm) for co-staining of CK-19 and desmin in mouse samples and semi-quantification for positive desmin area. Data are mean ± standard deviation. *n* = 3 for qPCR; *n* = 6–8 mice per group for hydroxyproline; *n* = 3 portal areas per mouse imaged from *n* = 8 mice per group for desmin. * *p* < 0.05, ** *p* < 0.01, *** *p* < 0.001, **** *p* < 0.0001.

We evaluated fibrosis by FG/SR staining and quantified hydroxyproline levels (a biochemical marker of collagen deposition), in liver samples. INVA8001 treatment resulted in an amelioration of hepatic fibrosis, as measured by decreased FG/SR staining and lowered hydroxyproline content, compared to untreated mice (Figures 5A,B). Furthermore, the gene expression levels of key fibrotic markers—*Col1a1*, *Timp2*, and *Tgfb1* in the liver were also significantly reduced following INVA8001 treatment (Figure 5C).

*Mdr2^−/−^* mice are characterized by the activation of HSCs and portal tract inflammation, both of which contribute to liver fibrosis. Desmin, a marker for HSC activation, showed strong immunoreactivity in *Mdr2^−/−^* mice livers, which was also significantly reduced with INVA8001 at a dose of 20 mg/kg, IP (Figure 5D).

### 3.5 INVA8001 prevents ductular reaction and biliary senescence

**Figure 6:**
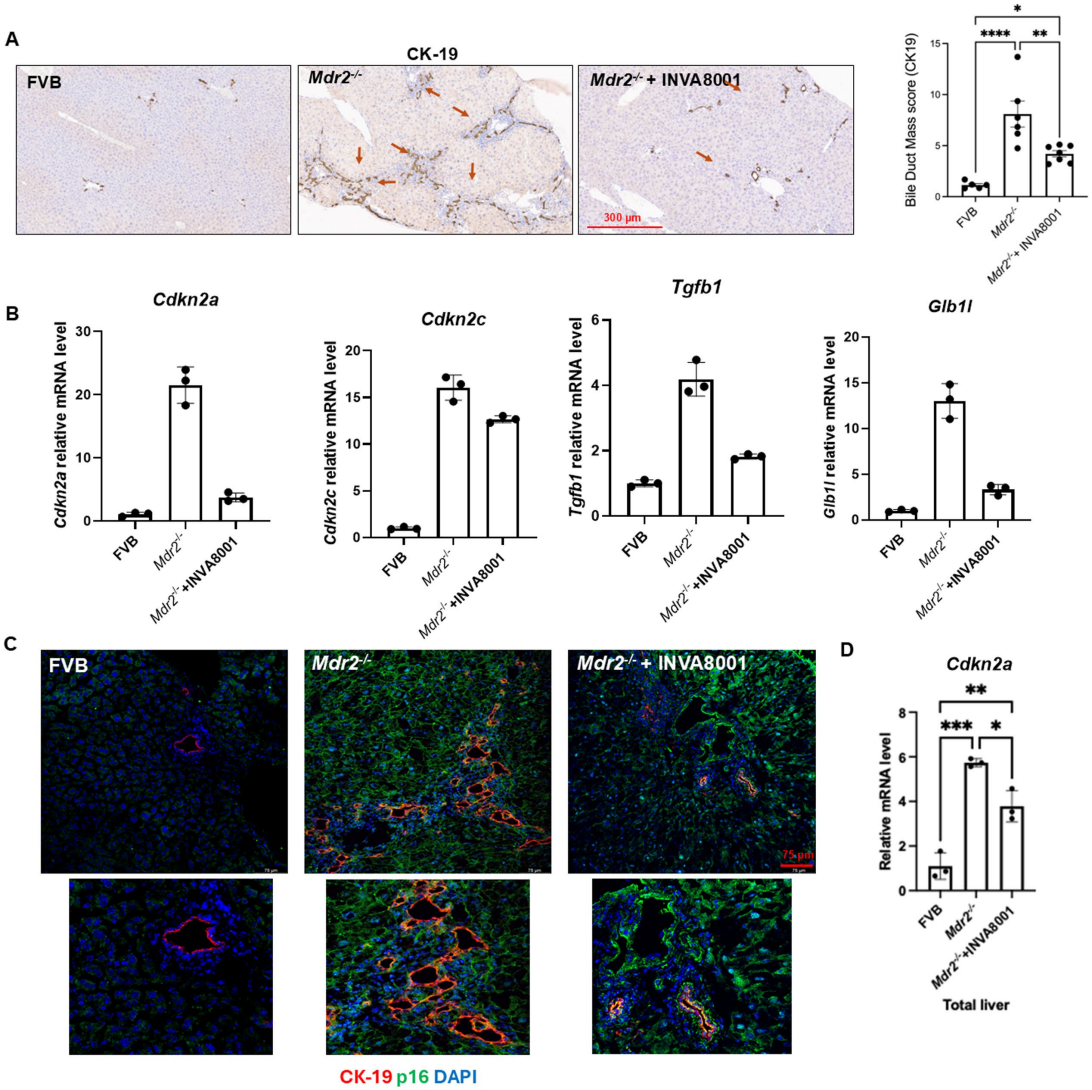
INVA8001 prevents ductular reaction and biliary senescence. **(A)** Staining (×10; scale bar, 300 μm) and semi-quantification of CK-19 in mouse samples; **(B)** qPCR for *Cdkn2a*, *Cdkn2c*, *Glb1l*, and *Tgfb1* in isolated cholangiocytes from mouse samples; **(C)** co-staining (×20; scale bar, 75 μm) for p16 and CK-19 in mouse samples; and **(D)** qPCR for *Cdkn2a* in total liver from mouse samples. Data are mean ± standard deviation. *n* = 4–5 portal areas per mouse imaged from *n* = 5–8 mice per group for CK-19; *n* = 3 reactions per group in total RNA isolated from isolated cholangiocytes or total liver from *n* = 8 mice per group; *n* = 3–5 portal areas imaged from *n* = 3–4 mice per group for p16/CK-19. * *p* < 0.05, ** *p* < 0.01, *** *p* < 0.001, **** *p* < 0.0001.

We measured the effect of blocking chymase activity on proliferating cholangiocytes, as well as senescence-associated markers. INVA8001 (20 mg/kg, IP) treatment led to a significant reduction in bile duct mass, as evidenced by a decrease in CK-19-positive bile ducts in *Mdr2^−/−^* mice compared to controls (Figure 6A). Furthermore, INVA8001 administration also reduced the expression of senescence-associated genes, such as *Cdkn2a*, *Cdkn2c*, *Tgfb1* and *Glb1l* in isolated cholangiocytes from *Mdr2^−/−^* mice (Figure 6B).

p16 (encoded by *Cdkn2a*) is a cyclin-dependent kinase inhibitor that regulates and slows down cell cycle progression. INVA8001 treatment reduced the level of p16 immunoreactivity and mRNA expression of Cdkn2a in the livers of *Mdr2^−/−^* mice at a dose of 20 mg/kg, IP (Figures 6C,D), representing a viable strategy to target these pathological processes in PSC.

## 4 Discussion

PSC is a rare, chronic, progressive cholestatic liver disease characterized by increased MC infiltration, inflammation, fibrosis, destruction of both intra- and extra-hepatic bile ducts, and eventual liver failure. Despite its clinical significance and increasing prevalence, effective pharmacological therapies for PSC remain lacking.

Chymase, an enzyme produced and released by MCs, plays a central role in the pathogenesis and progression of hepatobiliary diseases, including liver fibrosis, MASH, and PSC. Elevated hepatic chymase levels correlate with fibrosis severity, and the presence of chymase-positive MCs in fibrotic areas indicates their direct involvement in disease progression (16, 22). Functionally, chymase exerts both paracrine and autocrine effects, influencing inflammation, extracellular matrix remodeling, and local tissue signaling through its proteolytic activity. Although most effects are paracrine, autocrine loops further amplify MC activation and tissue injury (6, 23, 24).

In the present study, we evaluated the chymase pathway as a critical regulator of PSC pathogenesis and demonstrated that pharmacological inhibition of chymase using a selective small-molecule chymase inhibitor, INVA8001, is associated with improvements in disease-related features in a murine model of PSC. Our findings reveal that chymase levels are significantly elevated in liver biopsies from explanted end-stage PSC patients compared to healthy controls. These results are consistent with prior studies indicating increased infiltration of chymase- and tryptase-positive MCs in the fibrotic portal tracts of PSC livers (25, 26). INVA8001 suppressed this response, suggesting broader therapeutic effects than MC stabilization with cromolyn sodium in PSC-induced damage (27).

Previous studies have demonstrated that INVA8001 (ASB17061), administered at 10 mg/kg for 4 weeks in a model of aortic aneurysm, is effective and it was inferred that a dose exceeding 10 mg/kg could be used for increased efficacy (17). Accordingly, for this study, we used a dose of 20 mg/kg administered over a 2-week period in *Mdr2^−/−^* mice. INVA8001 treatment decreased the number of chymase- and tryptase-positive cells, and suppressed mRNA expression of MC markers such as *Fcer1a*, *Mcpt1*, and *Tpsb2*. These findings are supported by prior reports that chymase regulates MC proliferation and survival possibly through substrates like SCF and IL-33 (28–31). By interrupting this feed-forward loop, chymase inhibition may disrupt the chronic inflammatory environment that characterizes PSC.

Importantly, INVA8001 treatment ameliorated histological liver injury in *Mdr2^−/−^* mice, as evidenced by reduced lobular damage, necrosis, and portal inflammation. This was accompanied by decreased activation of HSCs, as shown by diminished desmin staining, and a marked reduction in macrophage infiltration, indicating broad immunomodulatory effects of chymase inhibition on the hepatic microenvironment. While liver enzyme levels were unchanged, INVA8001 treatment led to significant reductions in serum and hepatic bile acid levels, suggesting improvement in bile acid homeostasis. Although changes in key liver enzymes can be reliable markers in human disease, in the *Mdr2^−/−^* model, serum liver enzymes can fluctuate and often show poor correlation with the extent of biliary injury and fibrosis, reflecting the cholangiocyte-driven nature of PSC. This model is characterized by chronic cholangiocyte dysfunction driven by impaired phospholipid secretion and toxic bile composition, rather than acute hepatocellular injury, which likely contributes to the limited sensitivity of transaminases in this setting. In addition, alkaline phosphatase in mice lacks the liver specificity observed in humans, further limiting its interpretability as a biomarker of cholestatic injury in preclinical studies (32–35). As our study design focused on defined endpoints, transient fluctuations in circulating enzymes may not have been captured.

One of the most clinically relevant observations was the ability of INVA8001 to attenuate hepatic fibrosis, a hallmark characteristic of PSC progression. Chymase inhibition by INVA8001 reduced collagen deposition (measured by FG/SR staining and hydroxyproline content) and downregulated fibrotic genes including *Col1a1*, *Timp2*, and *Tgfb1*. Together, these results highlight the role of chymase in promoting hepatic inflammation and HSC activation. Our findings demonstrate the significant effects of INVA8001 and support its potential as a therapeutic agent to alleviate liver injury and immune cell infiltration in PSC. These effects are consistent with chymase’s known ability to activate pro-fibrotic mediators such as collagen and TGF-*β* and are in line with previous reports of chymase inhibitors reducing cholestatic liver injury and TGF-β (35, 36).

Ductular reaction and biliary senescence are major contributors to PSC pathology. In PSC, cholangiocyte response to genetic and environmental insults leads to a heterogeneous response with a subpopulation acquiring a proliferative and reactive phenotype, while another subpopulation undergoes senescence and growth arrest, contributing to hepatic inflammation, fibrogenesis and disease progression (37). The reduction in CK19-positive ductal mass and the suppression of senescence-associated genes (*Cdkn2a*, *Cdkn2c*, *Glb1l*, *Tgfb1*) with INVA8001 treatment highlight a role for chymase in regulating cholangiocyte dysfunction. Given that cellular senescence contributes to chronic inflammation and fibrosis these findings position chymase inhibition as a promising strategy to interrupt the cycle of cholangiocyte injury and maladaptive repair. These data support a hypothesis in which chymase inhibition reduces MC-driven inflammation, decreases recruitment of inflammatory mediators and attenuates both fibrogenic and cholangiopathic responses in PSC.

Collectively, our data support the conclusion that chymase plays a multifactorial role in the progression of PSC, affecting MC activation, inflammation, fibrosis, ductular reaction, and biliary senescence. INVA8001, a selective and potent chymase inhibitor, demonstrated consistent efficacy across various pathological features in *Mdr2^−/−^* mice, establishing a strong preclinical rationale for further development of chymase inhibitors as therapeutic agents in PSC and potentially other MC-related diseases.

Despite the promising findings, several limitations should be considered. First, while there were significant effects on fibrotic signaling (i.e., TGF-β), a comprehensive mechanistic dissection may be warranted in the future. Second, given known sex-specific differences in disease severity in this model, the generalizability of these findings and the specific effects of INVA8001 in females remains to be determined (38, 39). Third, no consistent changes in serum liver enzymes were observed, which may reflect the known temporal variability and limited sensitivity of these markers in murine cholestatic models when assessed at single timepoints. Despite these limitations, the robust effects observed across histologic and molecular endpoints support continued investigation of chymase inhibition as a therapeutic strategy in PSC and other MC–driven diseases.

INVA8001 is an orally available small molecule with the potential to serve as a safe and effective therapeutic option for PSC, an orphan disease, with no approved medical treatment. The current standard of care for these patients falls into three categories: medical, endoscopic, and surgical. Medical treatment is focused on the off-label use of ursodiol (ursodeoxycholic acid), which at high doses has been shown to worsen the clinical outcome of the disease, including making some patients ineligible for transplant. In addition, other drugs such as cholestyramine, antihistamines, rifampicin, opioid antagonists, and bisphosphonates, aim at providing symptomatic relief. Endoscopic therapy is primarily used to manage dominant strictures, and a liver transplant (surgical) is the only therapeutic option for patients for enhanced survival (40–43). Moreover, given the costs and limited organ availability from donor programs, it is reserved for those with end-stage liver disease only. Thus, there is a high unmet need for safe and effective medical options, which are disease-modifying, halt disease progression to cirrhosis, and delay or avoid liver transplant.

INVA8001 has been previously evaluated in a Phase 2 clinical trial (44) for atopic dermatitis and demonstrated a favorable safety and tolerability profile, with no significant adverse effects reported. This safety profile, along with a wide therapeutic window, supports further evaluation of INVA8001 in longer-term studies and in combination with approved or investigational antifibrotic therapies. The translational potential of INVA8001 may also be enhanced through biomarker-guided clinical trials in PSC, particularly in patients with MC–dominant or fibrotic phenotypes.

Beyond PSC, INVA8001 may have broader relevance in diseases characterized by MC activation and chymase-driven pathology, including chronic urticaria, atopic dermatitis, asthma, MASH, and eosinophilic gastrointestinal disorders. In summary, INVA8001 represents a promising therapeutic strategy for targeting MC–mediated disease processes; however, further studies are needed to define its long-term efficacy and clinical applicability.

## Data Availability

The original contributions presented in the study are included in the article/Supplementary material, further inquiries can be directed to the corresponding author.
