# Supplementary material for: INVA8001, a novel and highly selective chymase inhibitor, ameliorates liver inflammation, fibrosis, and hyperplasia in Mdr2 knockout mice
Source: Front Med (Lausanne). 2026 Jun 9;13:1840071. doi: 10.3389/fmed.2026.1840071 (PMC13288762; doi:10.3389/fmed.2026.1840071)
Supplement: Supplementary file 1 [file Data_Sheet_1.DOCX]

Supplemental Table 1 List of antibodies

| Antibodies | Application | Source | Catalog no. |
| --- | --- | --- | --- |
| CK-19 | IF | Developmental Studies Hybridoma Bank (Iowa City, IA) | TROMA-III |
| CK-19 | IHC | Abcam | ab52625 |
| Chymase (human) | IHC | Abcam | ab111239 |
| mMCP-1(mouse) | IHC | Santa Cruz | Sc-17041 |
| Tryptase (human) | IHC | Cell Marque Antibodies | 342M-15 |
| Tryptase (mouse) | IHC | LifeSpan BioSciences | LS-B15255 |
| Desmin | IF | R&D Systems | AF3844 |
| F4/80 | IHC | Cell Signaling | 70076S |
| p16 | IF | Abcam | ab189034 |

Supplemental Table 2 List of real-time PCR primers

| Gene | Species | Detected transcript | Source |
| --- | --- | --- | --- |
| Col1a1 | Mouse | NM_007742 | QIAGEN |
| Cdkn2a | Mouse | NM_010233 | QIAGEN |
| Cdkn1a | Mouse | NM_008084 | QIAGEN |
| Cdkn2c | Mouse | NM_007671 | QIAGEN |
| Fn1 | Mouse | NM_010233 | QIAGEN |
| Fcer1a | Mouse | NM_010184 | QIAGEN |
| Gapdh | Mouse | NM_008084 | QIAGEN |
| Glb1l | Mouse | NM_029010 | QIAGEN |
| Mcpt1 | Mouse | NM_008570 | QIAGEN |
| Tgfb1 | Mouse | NM_011577 | QIAGEN |
| Timp2 | Mouse | NM_011594 | QIAGEN |
| Tpsb2 | Mouse | NM_010781 | QIAGEN |
